# Phaeochromocytoma Crisis Presenting As Inverted Takotsubo Cardiomyopathy in a Young Woman: A Successful Outcome

**DOI:** 10.7759/cureus.116145

**Published:** 2026-09-12

**Authors:** Nitish Datta, Shinjini Mukherjee, Tom Hyde, Shad Khan, Auditi Naziat

**Affiliations:** 1 General Medicine, Great Western Hospitals NHS Foundation Trust, Swindon, GBR; 2 Cardiology, Great Western Hospitals NHS Foundation Trust, Swindon, GBR; 3 Endocrine Surgery, Oxford University Hospitals NHS Foundation Trust, Oxford, GBR; 4 Endocrinology and Diabetes, Great Western Hospitals NHS Foundation Trust, Swindon, GBR

**Keywords:** adrenalectomy, cardiogenic shock, catecholamine cardiomyopathy, inverted takotsubo cardiomyopathy, mechanical circulatory support, phaeochromocytoma crisis, reverse takotsubo syndrome, stress cardiomyopathy

## Abstract

Phaeochromocytoma crisis is a rare, life-threatening endocrine emergency characterised by profound haemodynamic instability and multiorgan dysfunction. We report the case of a previously healthy 31-year-old woman who presented with sudden-onset cardiopulmonary decompensation, marked blood-pressure variability, pulmonary oedema, and cardiogenic shock, with biochemical evidence of myocardial injury. Echocardiography demonstrated apical hypercontractility with akinesia of the basal and midventricular segments and a left ventricular ejection fraction of 28%, consistent with reverse takotsubo cardiomyopathy. Cross-sectional imaging revealed a large 9 cm left adrenal mass, which, together with markedly elevated plasma and urinary metanephrines, confirmed the diagnosis of phaeochromocytoma crisis. The patient required mechanical ventilation, vasoactive support, and intra-aortic balloon counterpulsation. Following successful resuscitation and stabilisation with α- and β-adrenergic blockade, she underwent elective laparoscopic left adrenalectomy. Histopathological examination confirmed a high-risk phaeochromocytoma, while genetic testing was negative. Postoperatively, metanephrine concentrations normalised and bi-ventricular cardiac function recovered completely. This case highlights the importance of considering phaeochromocytoma crisis in patients presenting with otherwise unexplained haemodynamic instability and stress cardiomyopathy. Early recognition, multidisciplinary critical care, appropriate adrenergic blockade, and timely definitive surgery are essential and can be lifesaving.

## Introduction

Catecholamine-secreting neuroendocrine tumours called phaeochromocytomas originate from the chromaffin cells of the adrenal medulla and are generally considered rare [1], having an incidence rate of 1.9 cases per million person-years [2], and up to 30% are found incidentally [3]. Phaeochromocytomas can be hereditary or sporadic, with a known gene mutation present in about one-fourth of the sporadic cases [4,5]. The typical symptoms include severe headaches, hypertension, perspiration and palpitations [6], but can be variable, mimicking other diseases. As catecholamine surges can be intermittent or continuous, hypertension, which occurs as a result of such surges, can present as paroxysmal or sustained, respectively [7]. As a secondary cause of hypertension, phaeochromocytomas are often investigated, but routine testing is not recommended due to their rarity, unless indicated by clinical suspicion [8]. Biochemical screening with plasma or urine metanephrines is suggested by endocrine societies in all suspected cases, followed by anatomical localisation using imaging studies [9].

About 7-18% of phaeochromocytoma cases present with phaeochromocytoma crisis [10,11], which is characterised by grave manifestations like hypertensive crisis and multiorgan failure [12]. Further classification involves two main types of crises: type A, which is a limited crisis characterised by haemodynamic instability and end-organ damage or dysfunction, and type B, which is an extensive crisis with sustained hypotension (shock) and multiorgan failure [13]. However, due to a lack of globally accepted diagnostic criteria, rarity and sudden onset, it has not been possible to systematically study and appropriately clarify phaeochromocytoma crisis [13]. The overall mortality rate associated with this condition, according to recent studies, is 13.8% [12].

Phaeochromocytoma-associated cardiomyopathy may express in three principal forms, which are hypertrophic cardiomyopathy, dilated cardiomyopathy, and takotsubo (stress-induced) cardiomyopathy [14]. Takotsubo or stress cardiomyopathy is a reversible, impermanent left ventricular abnormality, resembling myocardial infarction but in the absence of coronary artery disease [15], occurring due to local toxic concentrations of catecholamines in the cardiac muscle [16]. In typical takotsubo cardiomyopathy, echocardiography demonstrates left ventricular ballooning and apical akinesis [16]. However, in younger patients, akinesia can be seen in the basal and mid-ventricular segments, sparing the apex, due to a higher density of catecholamine receptors in these areas than at the apex, leading to an 'inverted' takotsubo cardiomyopathy appearance [17].

We share the case of a previously healthy 31-year-old female who presented with a phaeochromocytoma crisis and inverted (reverse) takotsubo cardiomyopathy.

## Case presentation

A 31-year-old woman presented to her general practitioner with a 12-month history of progressively worsening headaches, palpitations, hot flushes, and excessive sweating. She had a long-standing anxiety disorder, which had recently worsened. Clinical examination and electrocardiography were normal, and she was discharged with reassurance. Later that evening, she called emergency medical services because of worsening symptoms, acute dyspnoea, and pleuritic chest pain. Paramedics found her pale, clammy, and hypoxaemic, with marked haemodynamic lability.

In the emergency department, her heart rate fluctuated between 48 and 178 beats/min and her blood pressure between 95/54 and 178/111 mmHg. Her oxygen saturation was 89% despite 15L O2. She remained afebrile and alert. Chest auscultation revealed bibasal crepitations. Although the electrocardiogram recorded earlier by her general practitioner had been normal, recordings obtained by the paramedics and in the emergency department showed ST-segment depression. Arterial blood gas analysis demonstrated type 1 respiratory failure and severe lactic acidosis, with a pH of 7.226 (reference range: 7.350-7.450). Initial blood investigations showed marked leucocytosis, troponin I of 2,846 ng/L (reference range: 0-11.6 ng/L), D-dimer of 786 ng/mL (reference range: 0-500 ng/mL), and C-reactive protein of 6.0 mg/L (reference range: 0-5 mg/L). Thyroid function was normal. Chest radiography showed no acute abnormality.

Pulmonary embolism and chest infection were initially considered. Therapeutic low-molecular-weight heparin, intravenous fluids and broad-spectrum antibiotics were commenced, and urgent computed tomography pulmonary angiography (CTPA) was arranged with early intensive care involvement. In the radiology department, her condition deteriorated abruptly: oxygen saturation fell to 70% despite 15 L/min oxygen through a non-rebreather mask, and she developed pink frothy sputum and signs of circulatory compromise. She was transferred immediately to the intensive care unit.

CTPA excluded pulmonary embolism but demonstrated pulmonary oedema. It also identified an incidental complex cystic mass in the left upper quadrant, displacing the stomach and pancreatic tail (Figure 1). 

**Figure 1:**
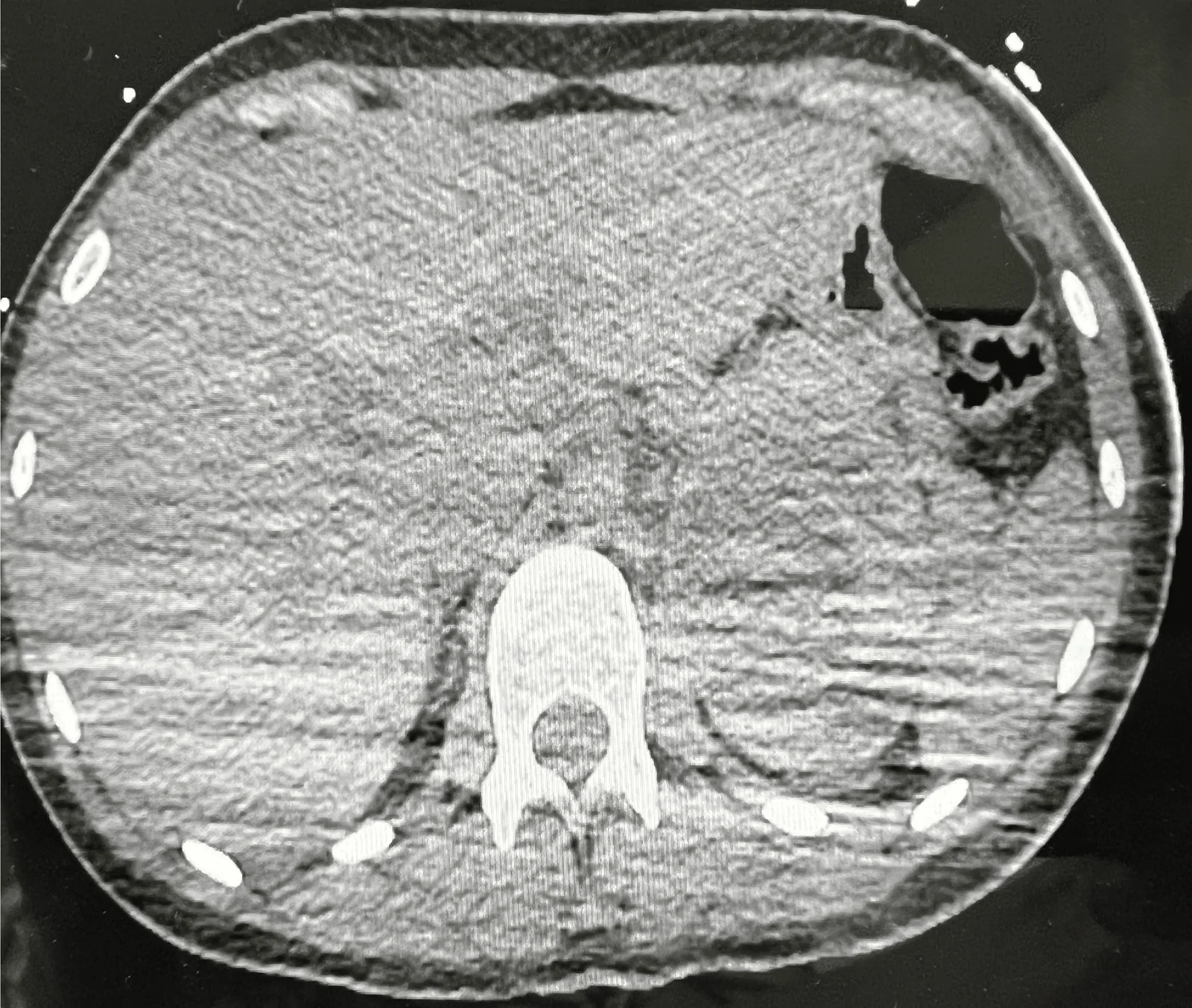
Computed tomography pulmonary angiography No evidence of pulmonary embolism. Extensive inflammatory changes in both lungs and features of atypical pneumonia. Incidental likely complex cystic lesion in the left upper quadrant, displacing the stomach and pancreatic tail, with impression of internal septations

Emergency endotracheal intubation was performed using propofol and fentanyl, and intravenous furosemide was administered for pulmonary oedema. After intubation, transient hypotension was followed by severe hypertension exceeding 250/150 mmHg and tachycardia to 150 beats/min. Repeated boluses of sedation were required to normalise her haemodynamic status. Her blood pressure continued to fluctuate between high and normal. Urgent bedside transthoracic echocardiography performed by the on-call cardiologist showed apical hypercontractility with akinesia of the basal and mid-ventricular segments and a left ventricular ejection fraction of 28%, consistent with reverse takotsubo cardiomyopathy (Figure 2).

**Figure 2:**
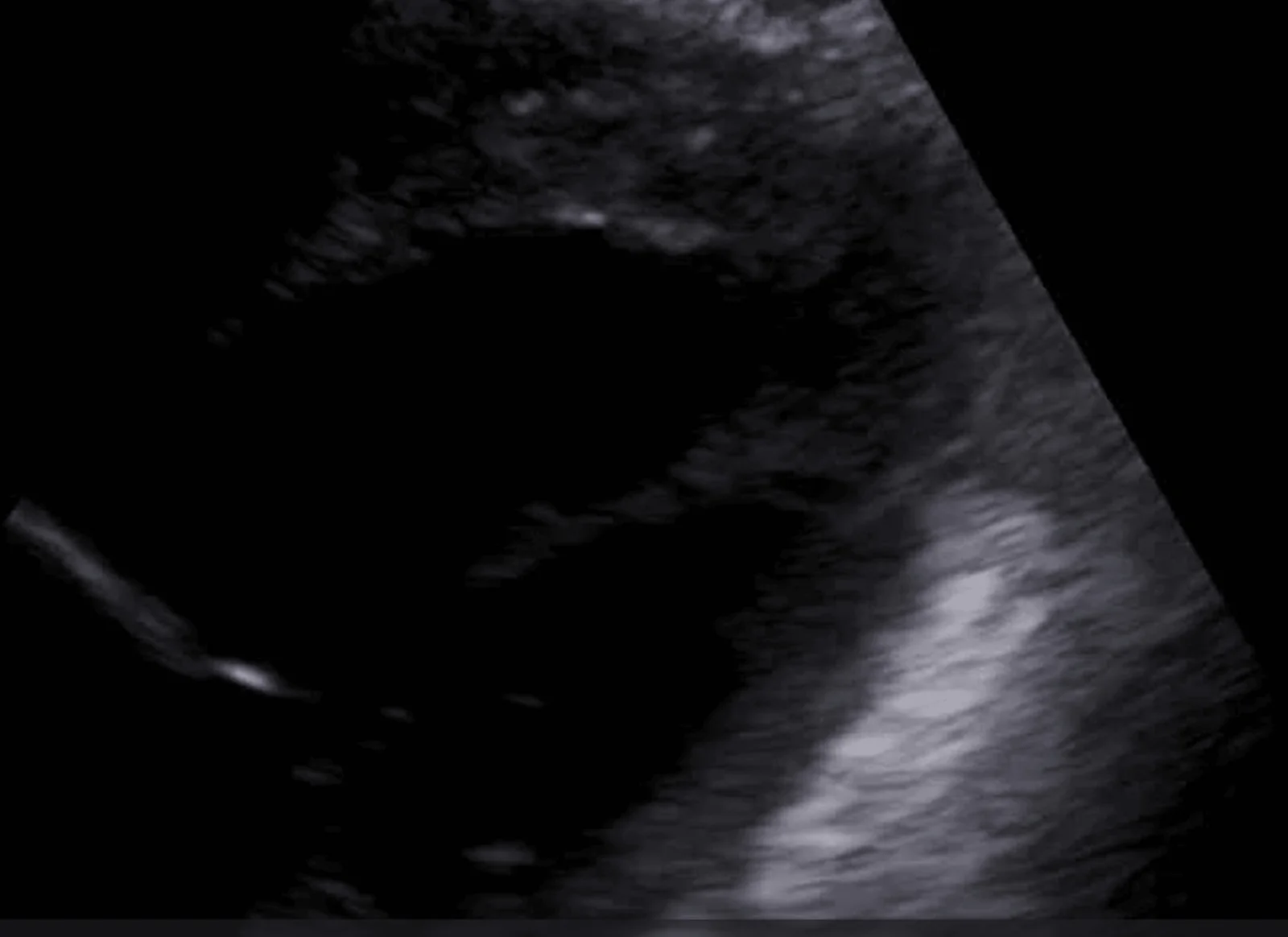
Echocardiography showing inverted takotsubo cardiomyopathy

In this clinical context, phaeochromocytoma became the leading diagnosis, and coronary angiography was deferred despite the marked troponin elevation. Blood samples were collected for plasma metanephrines. After initial stabilisation, abdominal computed tomography confirmed an 8.4 cm complex left adrenal mass radiologically consistent with phaeochromocytoma. A CT abdomen was arranged after stabilising the patient, which confirmed a complex large adrenal mass, measuring 8.4 centimetres, radiologically consistent with a phaeochromocytoma (Figure 3).

**Figure 3:**
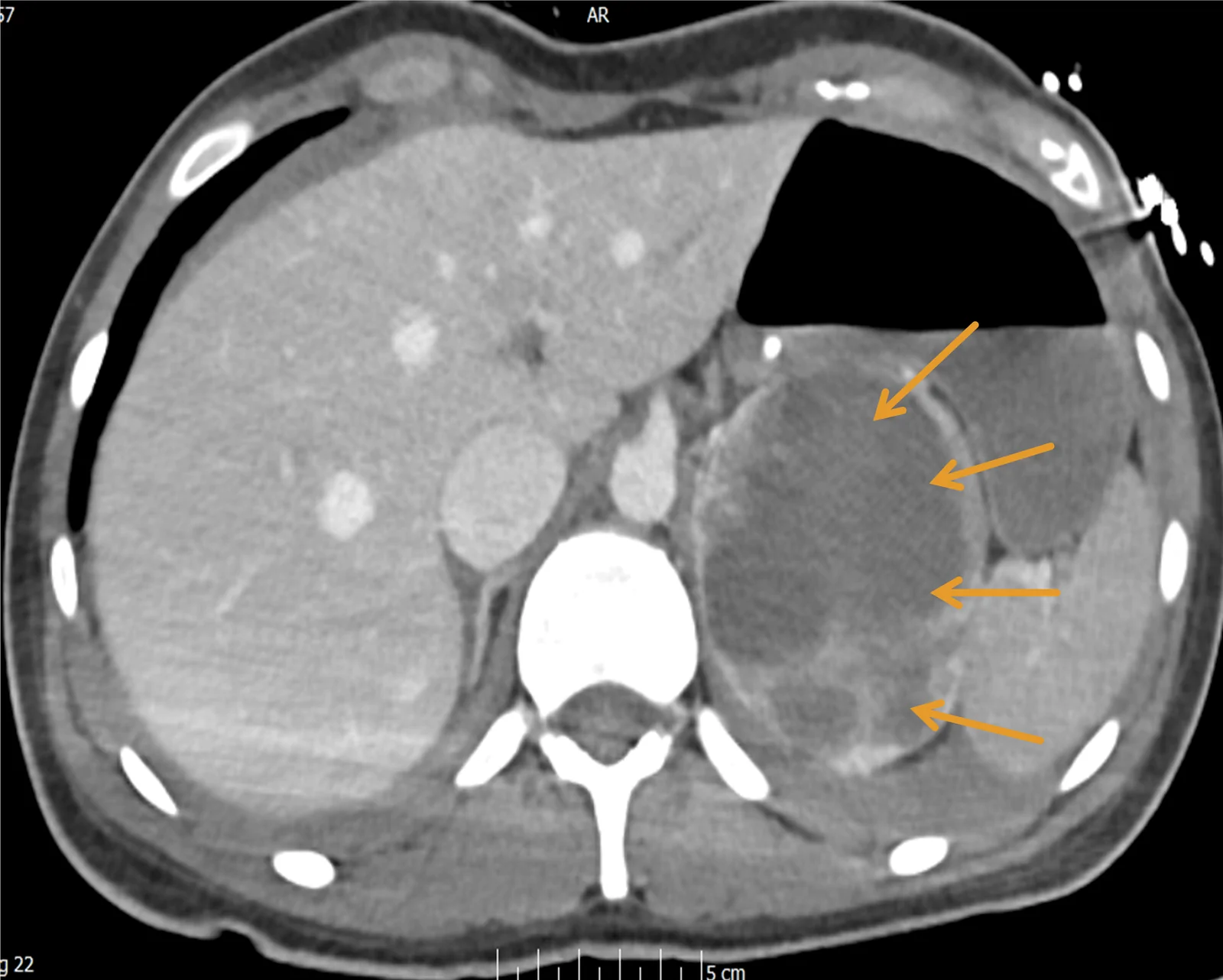
CT abdomen: complex large 8.4cm left adrenal tumour

Specialist endocrinology advice was obtained from a tertiary centre. Doxazosin was recommended if systolic blood pressure remained persistently above 160 mmHg because intravenous phentolamine was not immediately available in the hospital pharmacy.

That evening, her blood pressure was 180/120 mmHg, and a single 4 mg dose of doxazosin was administered through the nasogastric tube. Within hours, she developed persistent hypotension, with a blood pressure below 80/60 mmHg and a mean arterial pressure (MAP) of 50 mmHg, accompanied by tachycardia exceeding 160 beats/min. Further catecholaminergic inotropic agents were considered potentially hazardous, while aggressive fluid resuscitation was limited by the presence of pulmonary oedema and severe left ventricular dysfunction. A 500 mL bolus of 0.9% sodium chloride produced only transient haemodynamic improvement. The MAP subsequently fell to 40 mmHg, with a concurrent decline in urine output. Vasopressor support with metaraminol was therefore initiated. Its predominantly direct alpha-adrenergic vasoconstrictive action was considered less likely than that of agents with greater beta-adrenergic activity to aggravate tachycardia or increase myocardial oxygen demand. Despite this intervention, the hypotension persisted.

Transfer to a cardiothoracic centre for possible veno-arterial extracorporeal membrane oxygenation (ECMO) was planned; however, she was initially too unstable for transport. An intra-aortic balloon pump was therefore inserted, increasing the MAP to 80 mmHg and reducing the heart rate to 115 beats/min. She was safely transferred to the cardiothoracic centre and subsequently stabilised without ECMO. After extubation, recurrent hypertension was treated with a glyceryl trinitrate infusion for 24 hours. Phenoxybenzamine was then introduced, followed by bisoprolol, and cardiovascular support was weaned and discontinued five days after admission to the cardiothoracic centre. She was transferred to the nearby tertiary endocrine unit for optimisation of phaeochromocytoma management.

Biochemical investigations subsequently confirmed profound catecholamine excess (Table 1).

**Table 1:** Biochemical investigation results

Investigation	Result	Reference range
Plasma normetanephrine	>25,000 pmol/l	120-1180 pmol/L
24-hour urinary metanephrine	100.33 µmol/24 hr	0-1.4 µmol/24 hr
Normetanephrine concentrations	74 µmol/24 hr	0-3 µmol/24 hr

Dedicated adrenal computed tomography demonstrated a large left adrenal phaeochromocytoma with central haemorrhage. Metaiodobenzylguanidine scintigraphy (MIBG) confirmed avid uptake within the adrenal lesion and showed no evidence of metastatic disease (Figure 4).

**Figure 4:**
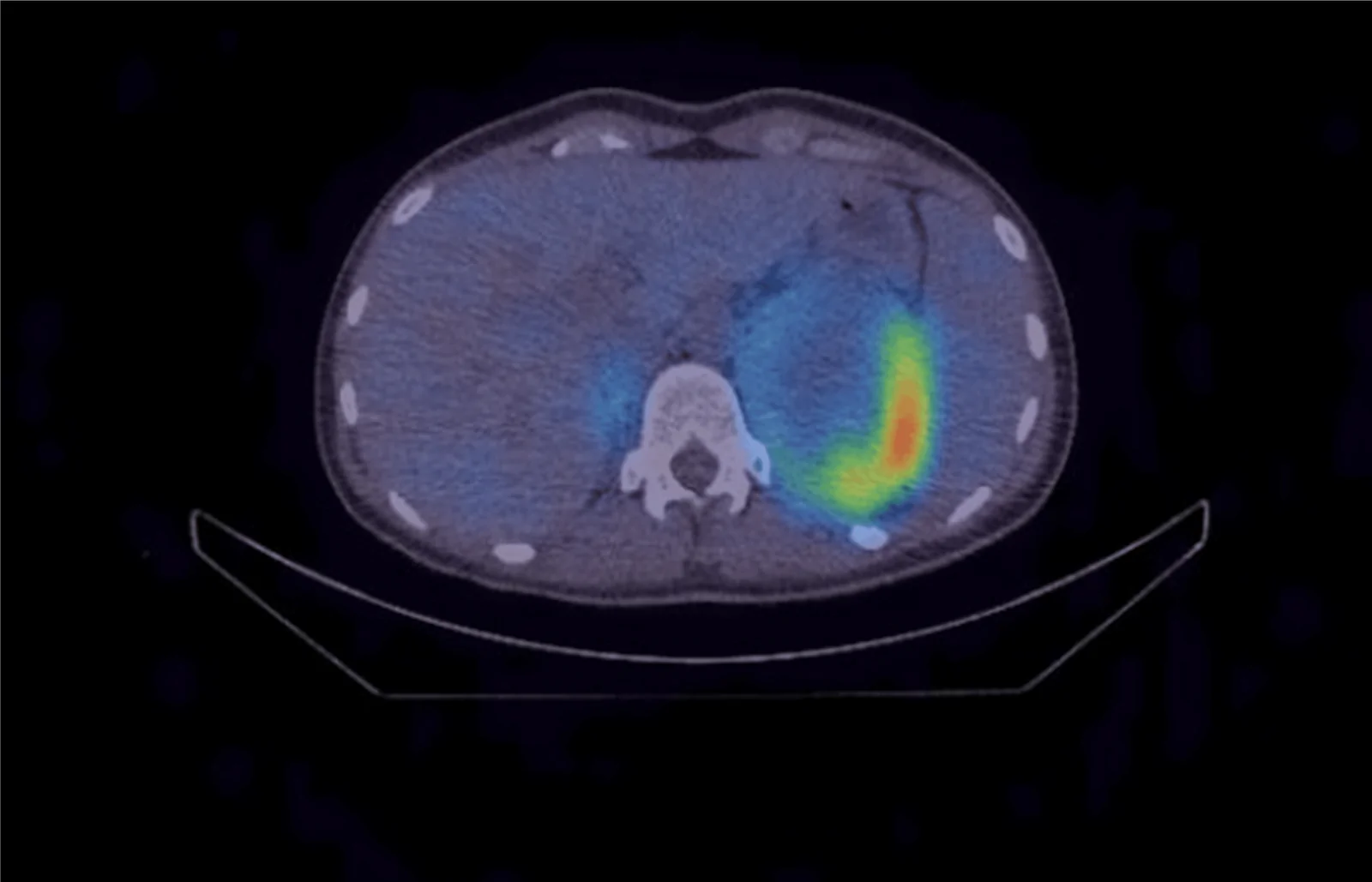
Metaiodobenzylguanidine scintigraphy scan Large phaeochromocytoma with central haemorrhage with no evidence of metastasis.

After three days in the endocrine unit, she was discharged home for elective left adrenalectomy and close follow-up by the local endocrine team.

She subsequently underwent elective laparoscopic left adrenalectomy. Splenomegaly was unexpectedly identified intra-operatively and, because of concern regarding bleeding risk, concurrent splenectomy was performed (Figure 5).

**Figure 5:**
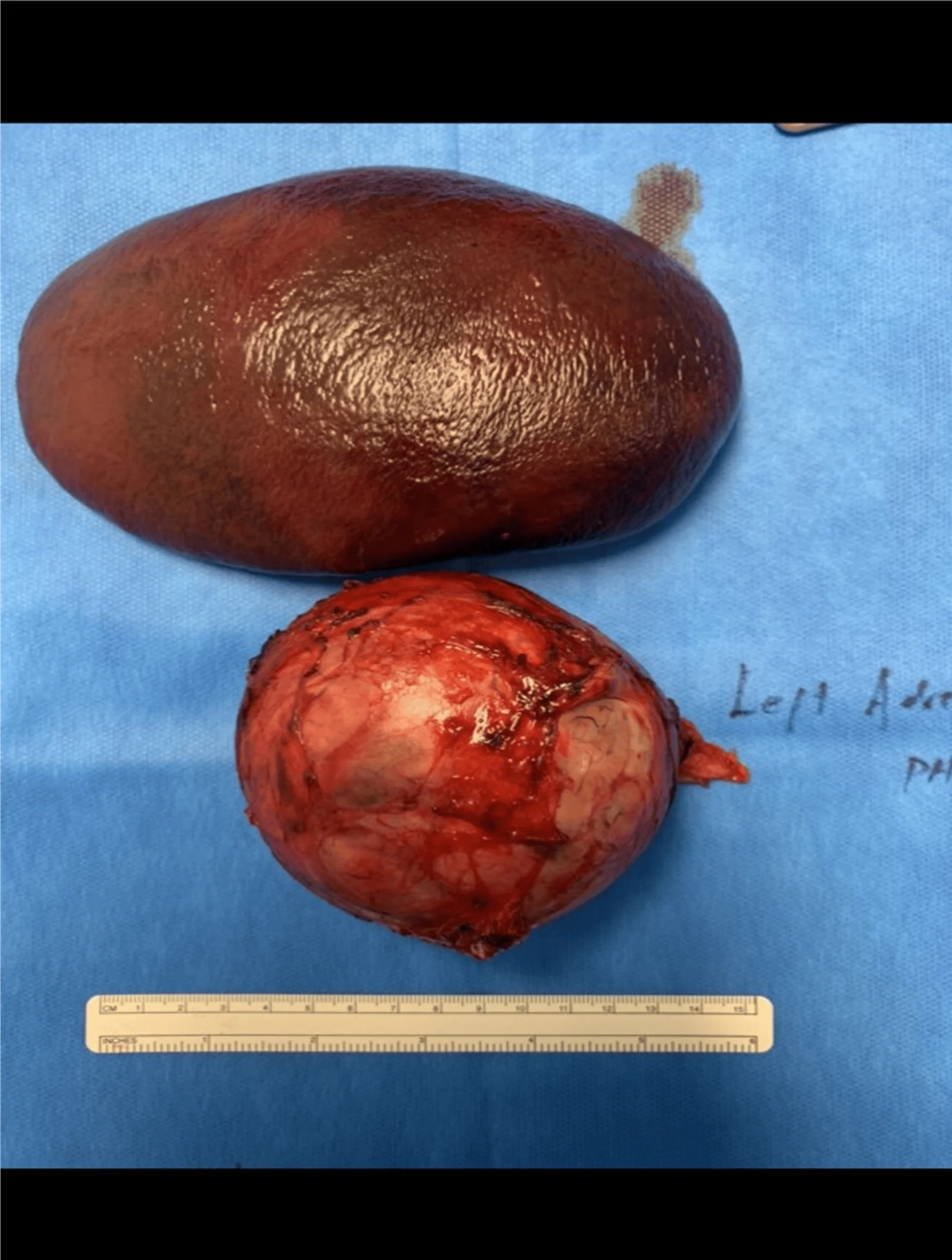
Resected adrenal tumour and large spleen

Post-operatively, plasma metanephrine concentrations normalised. Repeat echocardiography demonstrated complete resolution of the reverse Takotsubo pattern and restoration of normal bi-ventricular systolic function. Histopathological examination confirmed a phaeochromocytoma with high-risk histological features associated with an increased risk of recurrence and/or aggressive biological behaviour. Comprehensive molecular analysis did not identify pathogenic variants in FH, MAX, MEN1, RET, SDHA, SDHAF2, SDHB, SDHC, SDHD, TMEM127, or VHL genes.

Given the tumour size and adverse histological features, long-term biochemical and radiological surveillance was instituted. At the latest follow-up, she remained asymptomatic, with no evidence of biochemical or radiological recurrence.

## Discussion

The case report highlights the diagnostic challenge with regard to phaeochromocytoma and the need for a high index of clinical suspicion in atypical presentations. As phaeochromocytoma can mimic different conditions like pulmonary embolism, pneumonitis or other acute emergencies, there is a great chance of initial misdiagnosis, thus delaying life-saving critical interventions. Phaeochromocytoma diagnosis may also be missed during life, with certain autopsy studies showing 0.05-0.1% of patients having undiagnosed phaeochromocytomas [18]. Fortunately, in this case, the diagnosis was made within three hours of presentation.

A rapidly fluctuating blood pressure and circulatory collapse in this patient illustrate a type B phaeochromocytoma crisis. The case also illustrates that resuscitation of patients having a phaeochromocytoma crisis is always the priority, followed by treatment of associated conditions. Such patients might require intubation and vasoactive drug management [19]. Phaeochromocytoma crisis with profound hypotension or labile blood pressures, and cardiogenic shock with severity levels necessitating mechanical circulatory support is not common but increasingly recognised in the literature [20]. Instances of refractory shock secondary to catecholamine-induced cardiomyopathy, requiring intra-aortic balloon pump (IABP) support and/or veno-arterial extracorporeal membrane oxygenation (VA-ECMO), have been reported in patients [21-23]. A patient with fulminant catecholamine-induced cardiomyopathy mimicking acute myocardial infarction, who developed profound shock and was successfully bridged to recovery with VA-ECMO alone, was described by Suh et al. [21]. More recently, Zeng et al. [22] reported a case of severe phaeochromocytoma crisis with cardiogenic shock requiring combined VA-ECMO and IABP for circulatory stabilisation. Chen et al. [23] described a phaeochromocytoma crisis complicated by severe cardiogenic shock and respiratory failure in which ECMO support enabled definitive tumour resection and subsequent recovery. Takotsubo cardiomyopathy can be caused by a variety of factors that result in elevated catecholamine levels, amongst which an important cause is phaeochromocytoma [24]. Its first presentation may mimic an acute coronary syndrome [25]. In about 15% of cases of takotsubo cardiomyopathy, haemodynamic dysfunction requiring vasopressor drugs and/or an intra-aortic balloon pump is necessary, with an approximate 5% in-hospital mortality rate, primarily due to treatment-resistant cardiogenic shock or irreversible major co-existing medical conditions [26]. A takotsubo cardiomyopathy 'inverted' variant is characterised by apical segment contractility preservation or hyperkinesis, and basal and mid-ventricular segment hypokinesis or akinesis [27]. In a prospective study of 3265 patients presenting with troponin-positive acute coronary syndrome, out of 35 transient cardiomyopathy cases, 14 (40%) were found to have the inverted variant [28]. Several studies have shown an approximate prevalence range of 4 to 18% of stress cardiomyopathy in patients having phaeochromocytoma, and a few involving a crisis [10,29-31]. Our patient developed a reversible, inverted takotsubo cardiomyopathy on a background of phaeochromocytoma, most likely due to her young age, having more catecholamine receptors in the left ventricular base than the apex [32]. Although this case report has the limitation of showing phaeochromocytoma crisis with inverted takotsubo cardiomyopathy in just one patient, there are only a few such cases of phaeochromocytoma with inverted takotsubo cardiomyopathy that have been reported [33-35]. Jindal et al. [34] described a patient with cyclical blood pressure fluctuations and reverse takotsubo cardiomyopathy that led to the diagnosis of phaeochromocytoma. Similarly, Kimura et al. [35] reported inverted takotsubo cardiomyopathy associated with phaeochromocytoma and atypical electrocardiographic findings, further supporting the link between catecholamine surges and this distinctive contractile pattern. Phaeochromocytoma-associated inverse takotsubo cardiomyopathy has been linked to more complications [36], with the probable cause being right ventricular dysfunction associated with the inverted variant [37]. Certain cases also demonstrated haemodynamic collapse following intubation or induction of anaesthesia, suggesting that in patients with underlying catecholamine excess, sedation induced by vasodilation, positive pressure ventilation, reduced venous return, and an abrupt sympathetic tone alteration may precipitate cardiovascular decompensation [23,38]. The "paradox of hypotension" in phaeochromocytoma crisis, associated with high mortality, can be contributed to myocardial stunning by catecholamine and marked intravascular volume depletion, despite excessive circulating catecholamines [39]. Doxazosin, a selective alpha-1 blocker, used in pre-operative phaeochromocytoma management, is associated with fewer adverse haemodynamic effects than non-selective blockade with phenoxybenzamine [40,41]. However, patients with catecholamine-induced cardiomyopathy are particularly susceptible to severe hypotension following α-blockade initiation because of intravascular volume contraction, impaired myocardial function, and desensitised β-adrenoceptors [14]. Hence, intravenous non-selective α-blockade with phentolamine is often preferred due to its rapid titratability and ability to achieve immediate blood pressure control in such scenarios [42]. In the present case, the temporal association between doxazosin administration and refractory hypotension raises the possibility that α-blockade contributed to circulatory collapse by abruptly reversing catecholamine-mediated vasoconstriction in a patient already dependent on high systemic vascular resistance to maintain perfusion. However, the patient's preceding haemodynamic instability, pulmonary oedema, severe myocardial dysfunction, and post-intubation deterioration suggest that hypotension was more likely multifactorial, including reduced preload after initiation of positive pressure ventilation, catecholamine storm, myocardial dysfunction from reverse takotsubo cardiomyopathy, and impaired vascular responsiveness following catecholamine receptor downregulation rather than solely attributable to a single dose of doxazosin. Another important factor to consider is the presence of a possible tumour haemorrhage contributing to hypotension and acute shock, due to sudden catecholamine depletion after prior excessive release [13]. Among the differential haemodynamic effects of circulating catecholamines, dopamine-mediated resistant vasodilation at higher receptor activity thresholds may also paradoxically exacerbate hypotension in selected phaeochromocytoma phenotypes [43]. Identifiable mutations can be found in about 25% of sporadic phaeochromocytoma cases; hence genetic screening is recommended [44]. For high-risk phaeochromocytomas, lifelong follow-up is recommended with biochemical testing and imaging at regular intervals, as recurrence may occur even after complete resection [45]. As is being done in our case, structured surveillance is vital to detect recurrence early.

## Conclusions

Phaeochromocytoma, although rare, should remain an important differential diagnosis in patients presenting with unexplained haemodynamic instability, acute cardiopulmonary compromise, rapidly fluctuating blood pressures, or stress cardiomyopathy. This case also illustrates the considerable therapeutic challenges associated with phaeochromocytoma crisis, where both catecholamine excess and cardiovascular collapse may coexist. Successful management required a coordinated multidisciplinary approach involving intensive care, endocrinology, cardiology, anaesthetics, radiology, and surgery, together with careful haemodynamic support, temporary mechanical circulatory support, and definitive surgical resection.

Increased awareness of phaeochromocytoma crisis and its potentially catastrophic cardiovascular manifestations is essential to facilitate earlier diagnosis, minimise treatment delays, and improve patient outcomes in this life-threatening but potentially reversible condition.
